# Epstein Barr virus antigen-induced autoantibodies against complement C1q exacerbate renal disease in lupus-prone mice

**DOI:** 10.3389/fimmu.2026.1710424

**Published:** 2026-03-18

**Authors:** Eylul Tuncer, Solange Moll, Denise Dubler, Kristina Schulz, Marten Trendelenburg

**Affiliations:** 1 Clinical Immunology, Department of Biomedicine, University of Basel, Basel, Switzerland; 2 Division of Pathology, Department of Diagnostic, University Hospital of Geneva, Geneva, Switzerland; 3 Division of Internal Medicine, University Hospital Basel, Basel, Switzerland

**Keywords:** autoantibodies, complement C1q, Epstein–Barr virus, renal pathology, systemic lupus erythematosus

## Abstract

**Background and Aims:**

Systemic Lupus Erythematosus (SLE) is a complex autoimmune disease characterized by the development of autoantibodies against multiple antigens, including complement C1q, starter molecule of the classical pathway. Anti-C1q autoantibodies (anti-C1q) are not only a biomarker of disease activity but believed to contribute to the pathogenesis of proliferative lupus nephritis. Previous studies demonstrated that a key immunogenic site of C1q (so-called ‘A08’) shares an identical sequence with Epstein-Barr-Virus (EBV) Nuclear antigen-1, and that anti-C1q can be induced by this EBV antigenic site *in vivo*.

**Methods:**

We investigated whether an EBV-derived antigen can trigger a cross-reactive anti-C1q response in lupus-prone mice and enhance renal pathology. Mertk-deficient mice, which exhibit a defective clearance of apoptotic cells, were immunized with EBV-derived peptide. Antibody responses against the EBV antigen, intact C1q and the C1q-derived antigenic site A08 were determined, and renal pathology was assessed histologically and by electron microscopy.

**Results:**

The immunization with EBV antigen led to the generation of antibodies recognizing the C1q-derived antigen A08 in most, and the formation of anti-C1q with binding characteristics as occurring in SLE patients in a substantial subset of mice. Generation of anti-C1q was associated with accelerated mesangioproliferative glomerulonephritis and increased glomerular IgG and complement deposition.

**Conclusions:**

Our findings demonstrate that EBV-derived peptides can elicit pathogenic anti-C1q via molecular mimicry, thereby exacerbating renal disease in lupus-prone mice. The data provide mechanistic evidence for how an EBV antigen can accelerate SLE progression, and confirm the concept of anti-C1q being a driver of lupus nephritis.

## Graphical Abstract

**Figure:**
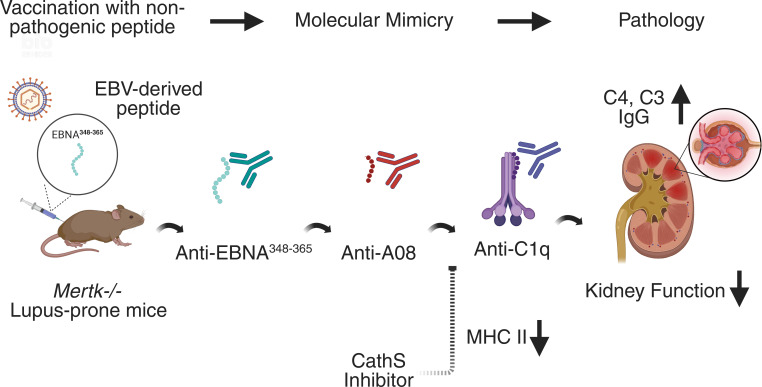

## 1 Introduction

Systemic Lupus Erythematosus (SLE) is a chronic autoimmune disorder characterized by the activation of the complement cascade and an aberrant production of a large repertoire of autoantibodies. Some of these autoantibodies can be used as markers of disease activity and can be detected up to a decade before disease onset (1). The pathogenic mechanisms leading to the development of SLE are still not well understood. The current view is that SLE is triggered and driven by a combination of genetic and environmental factors (2, 3). One of the strongest genetic risk factors for the development of SLE is homozygous deficiency of complement C1q (4), the starter molecule of the classical pathway (CP). Because C1q accelerates the clearance of apoptotic cells, C1q deficiency may lead to autoimmunity due to the accumulation of apoptotic cell debris causing prolonged exposure to the immune system which is believed to foster autoantibody formation (‘waste disposal hypothesis’) (5). Most patients with SLE do not have C1q deficiency, because C1q deficiency is extremely rare. However, a hallmark of SLE disease flares is activation of the classical pathway of complement as well as the deposition of C1q in affected tissues (6). In addition, a significant subset of SLE patients develops anti-C1q autoantibodies (anti-C1q) (7). The presence of anti-C1q is not only associated with disease activity but in particular with the occurrence of active lupus nephritis (7). Given these observations and the versatile functions of C1q (8), anti-C1q are likely to alter the function of the molecule and to play a pathogenic role in SLE. Because *in vitro* anti-C1q were shown to specifically recognize C1q bound to early apoptotic cells (9), the pathogenic effect of anti-C1q *in vivo* may be best seen in a disease model mimicking a situation with prolonged exposure to apoptotic cell debris as proposed by the ‘waste disposal hypothesis’.

In addition to a genetic predisposition, environmental factors such as viral infections appear to drive the autoimmune process in SLE. Here, the link between SLE and Epstein-Barr-Virus (EBV) is of particular interest, as previous EBV infection seems to be a prerequisite for the development of SLE (9, 10). In analogy to observations made in patients with Multiple Sclerosis (11) observational data demonstrate a nearly 100% seropositivity for EBV in adult SLE patients that exceeds the rate of seropositivity found in matched healthy controls (12, 13).

There are several hypotheses addressing the link between EBV infection and the development of SLE. One intriguing hypothesis suggests that autoantibodies as occurring in SLE are generated by molecular mimicry between EBV antigens and autoantigens. In this concept persistent cross-reactivity can disrupt immune tolerance over time, causing a positive feedback loop in which autoantibodies mediate cellular damage (14). This leads to the release of additional autoantigens, causing sustained immune activation. Previous studies demonstrated the cross-reactivity of anti-EBV nuclear antigen-1 (EBNA1) antibodies with known SLE autoantigens (13, 15–17). More recently, the hypothesis was supported by the finding that short linear mimicries of EBV are associated with autoantibody formation (18). However, specific antigenic sites leading to cross-reactivity are not well defined and functional data supporting the hypothesis of cross-reactivity between anti-EBV and the generation of pathogenic autoantibodies in SLE are limited. In addition, formation of anti-EBNA1 antibodies alone is not sufficient to disrupt the immune tolerance and drive autoimmunity, as more than 90% of individuals are exposed to EBV, yet the majority do not develop autoimmune disease (19). This suggests that additional genetic and/or genomic factors are needed for the development of SLE with the environmental challenge of EBV infection critically contributing to the disruption of the immune tolerance. In line with this view, SLE patients were shown to have a more diverse antibody response against the EBV-derived antigenic structure EBNA-1 than controls, including a more pronounced immune response against the C-terminal regions of EBNA-1 (20).

High affinity IgG anti-C1q autoantibodies from SLE patients predominantly target cryptic epitopes being located on the collagen-like region (CLR) of the C1q molecule (21). Our previous data demonstrate that C-terminal antigenic fragments of EBNA-1 of EBV (i.e. EBNA ^348-365^) can trigger the generation of such anti-C1q *in vivo* based on amino acid sequence identity between EBNA-1 at this site and a major linear epitope of C1q, the so-called ‘A08’ region (12). However, this induction was only achieved in C1q-deficient mice not carrying the target molecule. Therefore, the pathogenic role of EBV-induced anti-C1q remained to be determined.

To investigate this assumed role, we aimed at studying a model of autoimmune disease that is based on a well-defined defect in the clearance of apoptotic cells, i.e. *Mertk*-deficient mice. These mice, lacking the membrane tyrosine kinase c-mer (Mer receptor tyrosine kinase, Mertk), exhibit not only an impaired clearance of apoptotic cells but also develop progressive lupus-like autoimmunity being characterized by the production of IgG anti-dsDNA and mild mesangioproliferative glomerulonephritis (22). The model allowed us to address the following key questions: i) can anti-C1q as occurring in SLE patients be induced by an EBV-derived antigen in the context of a defective clearance of apoptotic cells, and ii) do the induced anti-C1q lead to disease acceleration in a manner attributable to their occurrence, thus confirming the pathogenicity of anti-C1q *in vivo.*

## 2 Materials and methods

### 2.1 Animal models and ethical compliance

Seven- to eight-week-old female *C1qa−/−* and *Mertk−/−* mice (C57BL/6J background, 19 ± 2 g) were used. The *C1qa−/−* strain was maintained for more than ten generations. *Mertk−/−* mice were kindly provided by Dr. Greg Lemke (The Salk Institute, USA) and bred in-house. Animals were group-housed in individually ventilated cages under standard conditions (18–23 °C, 40–60% humidity, 12-h light/dark cycle) with food and water *ad libitum*. All animal procedures complied with Swiss federal animal welfare regulations and were approved by the Cantonal Veterinary Office of Basel-Stadt and the Federal Food Safety and Veterinary Office (authorization no. 2633/33821 2982/36599).

### 2.2 Immunization protocol and CathS inhibitor treatment

To induce peptide-specific antibody responses, mice were immunized with synthetic peptides. EBNA^348–365^ (GSGGRRGRGRERARGGS) and the C1q-derived control peptide C08 (GAPGKDGYDGLPG) were synthesized at >95% purity (Peptide Elephant, Germany). Lyophilized peptides were reconstituted in PBS (Thermo Fisher Scientific, USA) at 2 mg/mL and emulsified 1:1 (v/v) with Complete Freund’s Adjuvant (CFA; InvivoGen, France) for primary immunizations. Mice were injected subcutaneously at the tail base with 100 µg peptide in 100 µL total volume. Boosters were administered on days 14, 28, and 42 using the same dose emulsified in Incomplete Freund’s Adjuvant (IFA; InvivoGen, France). Adjuvant-only mice received CFA for the primary immunization and IFA for booster injections, emulsified with PBS without peptide and administered using the same schedule and route as peptide-immunized animals. This group served as control for adjuvant-driven effects and to verify the peptide specificity of the antibody responses.

*Mertk−/−* mice (n = 15 per cohort) were immunized in three independent experiments, each following the prime-boost schedule. To assess the temporal effect of the Cathepsin S inhibitor anti-C1q IgG generation, *C1qa−/−* mice were used, as this strain reliably produces anti-C1q after EBNA^348–365^ immunization. Mice were divided into three treatment groups, each receiving Cathepsin S inhibitor RO5461111 (Roche, Switzerland) starting at different time points: day 2 post-immunization (d2 group, n = 15), day 15 after the first booster (d15 group, n = 45), or day 30 after the second booster (d30 group, n = 45). The inhibitor was delivered via chow admixture formulated by Research Diets Inc. (USA) to achieve ~30 mg/kg/day, based on average body weight and food consumption.

Mice were monitored for general health, body weight, and local inflammation. Blood was collected before immunization and prior to each booster. Serum was analyzed by ELISA for anti-EBNA348, anti-C08, anti-A08, anti-dsDNA, and anti-C1q antibodies. All experiments were performed in replicate cohorts to ensure reproducibility.

### 2.3 ELISA for anti-dsDNA, anti-peptide and anti-C1q antibodies

Mouse sera were thawed on ice, diluted in assay buffer (PBS, 1% BSA, 0.05% Tween-20; MyBioSource, Cat# MBS2600477), and assayed in duplicate on pre-coated 96-well plates. Anti-C1q IgG was measured by indirect ELISA on MaxiSorp, Nunc plates coated with 350 ng/well purified human C1q (Complement Technology). For peptide ELISAs, plates were coated with 500 ng/well NeutrAvidin (Pierce) followed by 500 ng/well biotinylated EBNA348, A08, or C08 peptides (Peptide Elephant). Sera were diluted 1:500 in PBS-T for peptide assays or 1:10 in high-salt buffer (0.5 M NaCl, 0.05% Tween-20) for C1q assays, incubated 1–1.5 h at RT, washed, and probed with HRP-conjugated anti-mouse IgG (Fcγ-specific; Sigma, Cat# SAB3701037, 1:20,000). Plates were developed with TMB (BD Biosciences), stopped with 2 M H_2_SO_4_, and absorbance measured at 450 nm (BioTek Synergy HTX, Agilent). Isotype/subclass-specific ELISA signals were detected using HRP-conjugated goat anti-mouse secondary antibodies, including anti-IgA (Invitrogen, Cat# 62-6720), anti-IgM (Invitrogen, Cat# 31440), anti-IgG1 (Invitrogen, Cat# A10551), anti-IgG2b (Novus, Cat# NBP2-68515), anti-IgG2c (Invitrogen, PA1-29288), and anti-IgG3 (Invitrogen, M32707).

For ELISA-based readouts, OD values were background-corrected (blank-subtracted) and animals were classified as antibody-positive (“responders”) or antibody-negative (“non-responders”) based on the distribution of negative biological controls (pre-immune sera and/or adjuvant-only controls) using a robust IQR-based threshold (e.g., values exceeding Q3 + 1.5×IQR of the negative controls for the respective assay). Known positive-control sera/mice were included to verify assay performance and inter-plate consistency. To assess sequence-specific inhibition of polyclonal anti-A08 antibody binding, immune sera were subjected to a solution-phase competition step prior to anti-A08 ELISA. Briefly, sera from EBNA^348–365^–immunized mice with detectable anti-A08 reactivity were diluted to a working concentration within the linear range of the assay and pre-incubated with increasing concentrations of soluble A08 peptide or a scrambled A08 control peptide (Peptide Elephant) for 30–60 min at room temperature (or 37 °C) with gentle agitation. Following pre-incubation, the mixtures were transferred to A08-coated ELISA plates and residual anti-A08 IgG binding was quantified using the standard anti-A08 ELISA protocol (HRP-conjugated anti-mouse IgG and TMB development). OD450 values were blank-subtracted and normalized to the no-competitor condition (set to 100%) to calculate percent residual binding (or percent inhibition).

### 2.4 Immunofluorescence staining of glomerular complement C4, C3, and IgG

Mouse kidneys were harvested and rinsed in ice-cold PBS (Thermo Fisher) to remove blood, embedded in OCT and snap-frozen in liquid nitrogen (Air Liquide, Zürich), and stored at −80°C. Cryosections (8–10 µm) were prepared (CM3050S, Leica) and mounted on Superfrost Plus slides (Thermo Fisher, USA). Sections were thawed (30 min, RT), washed in PBS/0.05% Tween-20, and blocked (30 min) in PBS-Tween with 1% BSA and 1% FCS. Primary antibodies were incubated overnight at 4 °C: rabbit anti-mouse C3 (Abcam, UK 1:100, [11H9] Cat# ab11862), rabbit anti-mouse C4 (Abcam, 1:100, Cat# ab322106), and goat anti-mouse IgG (Jackson, USA Cat# 115-005-003, 1:200). After three washes, sections were incubated (1 h, RT, dark) with secondary antibodies (1:500): Alexa Fluor 488 donkey anti-goat IgG Cat# A-11055 and Alexa Fluor 647 goat anti-rabbit IgG Cat# A-21244 (Invitrogen). Nuclei were counterstained with DAPI (0.5 µg/mL) during mounting in Fluoroshield Antifade. Images were acquired on a Nikon Ti2 microscope with 4× and 20× Plan Apo objectives. Quantification was performed in ImageJ on 10–15 glomeruli per section using ROIs defined by nuclear/morphological features. Mean fluorescence intensity per glomerulus was averaged per sample.

### 2.5 Serum creatinine and blood urea nitrogen measurement

Serum creatinine and BUN concentrations were measured using enzymatic colorimetric assays according to manufacturers’ protocols. Creatinine was quantified with the Mouse Creatinine Assay Kit (Crystal Chem, Cat# 80350, USA), which employs a two-step enzymatic reaction with sarcosine oxidase and peroxidase. Briefly, 8 µL of serum was incubated with 270 µL of reaction reagent at 37 °C for 5 min, followed by an initial absorbance reading at 550 nm on a microplate reader (BioTek, USA). Then, 90 µL of peroxidase solution was added, and a second 5 min incubation was performed before final absorbance measurement. Creatinine concentrations were calculated against a standard curve (0.15–13.5 mg/dL) and reported as the mean of technical duplicates. BUN was quantified from the same serum samples using the QuantiChrom™ Urea Assay Kit (BioAssay Systems, Cat# DIUR-100, USA).

### 2.6 Histological staining

Paraffin-embedded kidney tissues were sectioned at 5 ;µm (Leica RM2235), mounted on Superfrost Plus slides (Thermo Fisher Scientific), deparaffinized in xylene, and rehydrated through graded ethanol. For H&E staining, sections were incubated in Mayer’s hematoxylin (Sigma-Aldrich) for 5 ;min, differentiated in 1% acid alcohol, and counterstained with eosin Y (Sigma-Aldrich) for 1 ;min. PAS staining was performed using 0.5% periodic acid (Merck) for 10 ;min followed by Schiff reagent (Sigma-Aldrich) for 15 ;min; sections were counterstained with hematoxylin. For Gomori’s methenamine silver (sFOG) staining, sections were incubated in preheated methenamine silver solution (Bio-Optica, 04-130802) at 60 ;°C for 20 ;min, toned with 0.1% gold chloride (Electron Microscopy Sciences), fixed in 5% sodium thiosulfate (Merck), and counterstained with light Green SF Yellowish (Sigma-Aldrich, USA). Slides were dehydrated, cleared, and mounted in Eukitt mounting medium (Sigma-Aldrich, USA), and imaged using a Nikon Eclipse Ti2 widefield microscope with Plan Apo 20× and 40× objectives.

### 2.7 Elution of IgG from mouse kidneys

Mouse kidneys were harvested and rinsed in ice-cold PBS (Thermo Fisher) to remove blood, snap-frozen in liquid nitrogen (Air Liquide, Zürich), and stored at −80 °C. For IgG extraction, kidneys were thawed on ice and homogenized in lysis buffer (50 mM Tris-HCl, pH 7.4; 150 mM NaCl; 1% Nonidet P-40; Sigma-Aldrich) supplemented with protease inhibitors (Roche Complete Mini, Basel). Homogenates were incubated on a rotator (4 °C, 1 h), centrifuged at 20,000 × g (30 min, 4 °C; Eppendorf), and supernatants were filtered (0.45 µm, Millipore). Protein concentration was measured using the BCA assay (Thermo Fisher). For IgG purification, clarified lysates were applied to a Protein G Sepharose column (GE Healthcare, USA). Eluted fractions were pooled, concentrated with Amicon Ultra centrifugal filters (Millipore), and quantified at 280 nm using a Nanodrop spectrophotometer (Thermo Fisher).

### 2.8 Transmission electron microscopy of mouse glomeruli

Mouse kidneys were immersion-fixed in 1% Paraformaldehyde in 0.1 M PIPES buffer (pH 7.4) at 4 °C, rinsed, and post-fixed in 1% Osmium tetroxide for 1h. Samples were dehydrated through graded ethanol, transitioned through propylene oxide, and embedded in Epon 812 epoxy resin. Ultrathin (~70 nm) sections were cut with an EM UC7 ultramicrotome, mounted on formvar-coated copper grids, and stained with 2% Uranyl acetate followed by Reynolds’ lead citrate. Imaging was performed on a Talos L120C transmission electron microscope (Thermo Fisher) at 120 kV with a 4k × 4k CMOS camera. Multiple glomeruli were analyzed per sample to assess podocyte foot processes, glomerular basement membrane integrity, and endothelial fenestrations.

### 2.9 Statistical analysis

All statistical analyses were performed using GraphPad Prism 10. Data are shown as individual biological replicates with summary statistics as indicated in the figure legends (mean ± SD/SEM or median with interquartile range). All tests were two-tailed, and p < 0.05 was considered statistically significant. For ELISA-based readouts, animals were classified as antibody-positive (“responders”) or antibody-negative (“non-responders”) using a predefined positivity threshold based on negative controls (e.g., adjuvant-only controls and/or pre-immune sera), applied consistently within each assay. Comparisons of proportions between independent groups (e.g., frequency of antibody-positive animals, distribution of histological categories) were analyzed using Fisher’s exact test when expected counts were small; otherwise χ² tests were used. For paired/dependent categorical comparisons (same animals classified under two conditions), McNemar’s test was applied when applicable. For normally distributed continuous variables, two-group comparisons used unpaired t-tests and multi-group comparisons used one-way ANOVA with appropriate *post-hoc* correction. If normality was not met or sample size was small, non-parametric tests were used (Mann–Whitney U for two groups, Kruskal–Wallis for ≥3 groups with Dunn’s *post-hoc* correction). Longitudinal measurements across multiple bleedings were analyzed using repeated-measures approaches (RM-ANOVA for parametric data or Friedman test/mixed-effects models for non-parametric data or missing values), with multiple-comparison correction as appropriate. Where multiple pairwise comparisons were performed, p values were adjusted using Tukey’s (parametric) or Dunn’s (non-parametric) procedures as appropriate.

## 3 Results

### 3.1 Anti-C1q can be induced by an EBV-derived antigenic site on a lupus-prone genetic background

**Figure 1:**
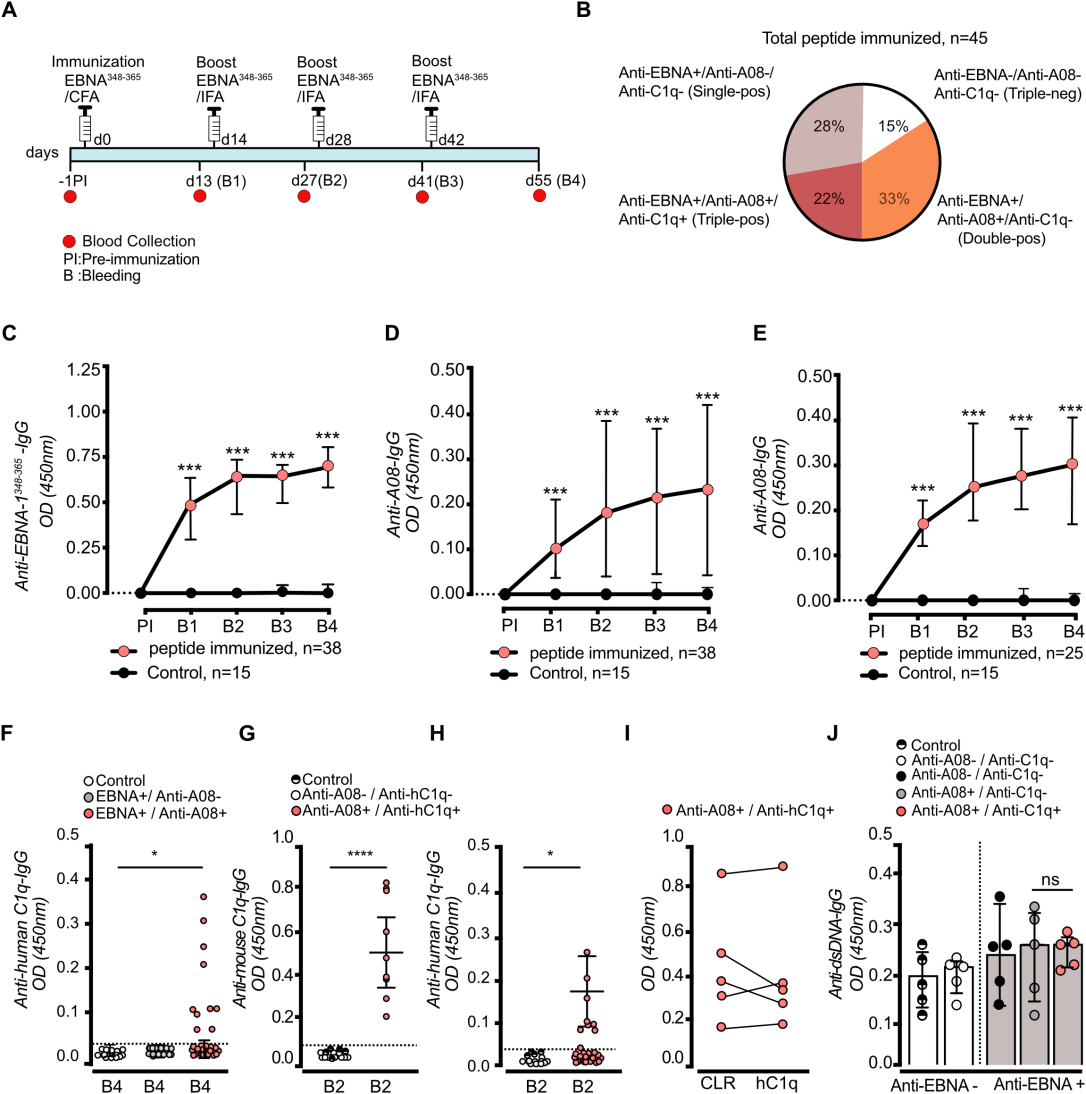
Immunization with EBNA^348–365^ peptide induces anti-A08 and anti-C1q autoantibodies in *Mertk-/-* mice. 7–8 weeks-old female Mertk-/- mice were immunized with EBNA^348–365^ peptide in CFA on day 0 (d0) and received subsequent boosts in IFA as outlined in the schematic **(A)**. Frequency distribution of serologically distinct subgroups based on status of three antibodies (anti-EBNA^348-365^, anti-A08, and anti-C1q) at B4 are shown. Triple-positive mice constituted 22%, while single- and double-positive subsets were also detected **(B)**. Blood was collected at baseline [pre-immunization (PI)] and at two-week intervals post-immunization (B1–B4). All antibody measurements were performed by ELISA. Serum anti-EBNA^348–365^ IgG titers increased significantly in immunized mice over time (in n = 38/45 immunized mice) compared with adjuvant-only controls (n = 0/15) **(C)**. Immunization also led to a progressive increase in anti-A08 IgG titers **(D)**, with a subset of 25 mice developing detectable responses **(E)**. By time point B4, 10 of the EBNA^348–365^ immunized mice developed both anti-A08 and anti-human C1q IgG **(F)**. Notably, these triple-positive mice also exhibited significantly elevated serum anti-mouse C1q IgG titers at B2 compared to B2 controls **(G)**. In comparison, the corresponding anti-human C1q IgG levels at time point B2 are shown **(H)**. Serum from anti-A08+/anti-C1q+ mice did not only recognize human C1q but also the collagen-like region (CLR) of C1q **(I)**. Anti-dsDNA IgG titers in anti-A08+/anti-C1q+ mice did not increase when compared to anti-C1q negative mice **(J)**. Data were plotted as median with interquartile range (IQR) **(C–E, J)**. Data were shown as dot plots with mean ± SD **(F–H)**. Significant differences were determined using two-tailed unpaired t-test or ANOVA for multiple comparisons; ns: non-significant, *P < 0.05, **P < 0.01, ***P < 0.001.

To explore the question, whether anti-C1q can be induced in a murine model of SLE, and to analyze the *in vivo* effect of induced anti-C1q, we subcutaneously immunized 7–8 weeks old female *Mertk-/-* mice (n = 45) with EBNA^348–365^ peptide. EBNA^348–365^ is a short 17 amino-acid long sequence of EBNA with sequence identity to A08, a C1q-derived epitope known as target for anti-C1q. The immunization followed a biweekly protocol (d0/d14/d28/d42) with accompanying blood sampling the day prior to the next immunization (**Figure 1A**). To assess the efficacy of the immunization, we first performed an ELISA using the EBNA^348–365^ peptide as the target. Additionally, an A08-peptide ELISA was conducted to evaluate the assumed cross-reactivity with the C1q-derived A08 epitope. Among 45 immunized mice, 38 (84.4%) mice exhibited an antibody response against EBNA^348-365^ (anti-EBNA^348-365^-IgG) (**Figures 1B, C**) and concurrently 25 mice demonstrated an additional immune response against the C1q-derived A08 epitope (anti-A08-IgG) (**Figures 1D, E**). The response was highly significantly different when compared to the CFA/IFA-adjuvant control group that received the adjuvants without peptide (n = 15) and that exhibited no detectable antibody response against EBNA^348–365^ confirming the specificity of the immunization-induced response (**Figures 1C–E**). Adjuvant-only (CFA/IFA) control mice did not develop detectable IgG responses against EBNA^348–365^, A08, or intact C1q, confirming that the observed antibody responses were peptide-specific. However, consistent with our previous findings in C1q-deficient mice, the OD values for A08-specific antibodies were relatively low, most likely reflecting the early, low-affinity nature of responses typically induced by peptide immunization in the absence of a strong adjuvant. The inclusion of mice not responding to the immunization with anti-EBNA^348–365^ peptide did not alter the statistical significance of the anti-EBNA^348–365^ IgG and anti-A08 IgG responses (p < 0.001, two-tailed Mann–Whitney, respectively; **Supplementary Figures 1B, C**). To assess whether anti-C1q antibodies were induced alongside the anti-EBNA^348–365^ response, we then performed an anti-humanC1q ELISA using sera collected from both immunized and control mice at the end point of the experimental setup (B4; Final Bleeding) (**Figure 1F**). Indeed, in the subset of anti-EBNA^348–365^ and anti-A08 double-positive mice (n = 25), 10 animals also developed anti-C1q antibodies. Thus, in total out of the 45 immunized mice, 7 mice (15%) did not respond to the immunization procedure. Another 13 mice (28%) remained single positive for anti-EBNA^348-365^, and 25 mice (33%) additionally became positive for anti-A08 IgG of which 10 mice (22%) were triple positive for anti-EBNA^348-365^, anti-A08 and IgG anti-C1q (**Figure 1B**). This fraction could be confirmed respectively was slightly higher in a subsequent experiment designed for the detailed histological analysis involving again 45 mice (triple positive n= 15/45, 33.3%). Responder frequencies (antibody-positive vs antibody-negative) were analyzed as categorical variables using Fisher’s exact test (or χ² where appropriate), in addition to comparisons of OD values.

To further assess the specificity of the EBNA^348–365^-induced anti-C1q antibodies, *Mertk-/-* mice were immunized with the C1q C chain-derived peptide C08 (GAPGKDGYDGLPG) following the same immunization protocol. Although a peptide-specific IgG response against C08 was detected these animals did not generate antibodies against intact C1q in spite of C08 being a C1q-derived peptide sequence (**Supplementary Figure 1C**). In addition, CFA/IFA-adjuvant controls (n = 15) developed neither IgG anti-A08 nor IgG anti-C1q responses. These findings confirm that the generation of anti-C1q antibodies after immunization with EBNA^348–365^ was peptide-specific.

Overall, anti-C1q titres began to emerge around day 27 (B2), following the second boost (**Figure 1F**). The initial ELISA analyses were performed using human C1q as the antigen (**Figures 1F, G**). Mouse C1q as antigen was subsequently included in the analysis to assess the pathogenic relevance within our mouse model. Given the high sequence homology between human and mouse C1q (75% identity in the C1q A chain), a substantial overlap in antibody reactivity was expected. Indeed, similar IgG binding was observed in both anti-humanC1q and anti-mouseC1q ELISAs. Notably, OD values were consistently higher in the anti-mouse C1q ELISA than in the anti-human C1q ELISA (**Figures 1F, G**; two-tailed Mann–Whitney U test, p < 0.0001). Both assays were run under high-salt conditions to increase stringency and reduce low-avidity binding. As an additional functional readout for sequence-specific binding within the polyclonal response, we performed a solution-phase competition assay for anti-A08 antibodies: pre-incubation of immune sera with increasing A08 peptide concentrations, but not scrambled control peptide, reduced residual anti-A08 ELISA binding in a concentration-dependent manner (**Supplementary Figure 1E**). Normalizing OD_405_ values to the no-competitor condition (OD_0_ = 0.456) yielded free antibody fractions (OD_i_/OD_0_), and log-linear interpolation between 3 and 10 µg/mL soluble A08 (free fractions 0.831 and 0.411, respectively) gave an apparent IC_50_ of ~7.8 µg/mL. Using a peptide molecular weight of 1428 g/mol, this corresponds to ~5.4 × 10–^6^ M, consistent with an average intermediate affinity of the polyclonal anti-A08 response (23).

Finally, IgG subclass profiling demonstrated that anti-C1q-reactive responder sera were dominated by IgG2c/IgG2b (and IgG3), whereas IgG1 signals were comparatively low (**Supplementary Figure 1D**), consistent with a class-switched anti-C1q response with a distinct subclass distribution.

Next, to identify the binding site of EBNA^348–365^–induced anti-C1q IgG, we performed an ELISA using the collagen-like region (CLR) of human C1q, as the assumed target structure of cross-reacting antibodies (i.e. A08) is located on the N-terminal CLR. As shown in **Figure 1H**, all anti-C1q autoantibodies also recognized the CLR of C1q, confirming that EBNA^348–365^ peptide-induced anti-C1q autoantibodies in our mouse model exhibited characteristics as observed for anti-C1q of human SLE patients (24).

To confirm the specificity of the anti-C1q response to the EBNA^348–365^ immunization we evaluated levels of anti-dsDNA autoantibodies. IgG anti-dsDNA antibodies develop in *Mertk*-/- mice over time as part of the autoimmune process. Comparing immunized and non-immunized groups, no significant differences in anti-dsDNA levels were observed. Additionally, there was no difference in anti-dsDNA titers between anti-C1q IgG positive and anti-C1q IgG negative mice in the EBNA^348–365^ IgG and A08 IgG double-positive cohorts, confirming the specificity of the anti-C1q IgG response after EBNA^348–365^ immunization (**Figure 1J**).

### 3.2 Application of a Cathepsin S inhibitor suggests T cell involvement in anti-C1q induction

We hypothesized that T-cell help is pivotal for anti-C1q autoantibody generation in *Mertk* -deficient mice in the process of molecular mimicry. To explore the mechanism, both EBNA ^348–365^ immunized and non-immunized *Mertk* -deficient mice were treated with a Cathepsin S (CathS) inhibitor (RO5461111) which down-regulates antigen presentation within antigen presenting cells (APCs) (25) The inhibition of CathS using this highly selective oral inhibitor was previously shown to have significant *in vivo* efficacy effectively mitigating lupus-like autoimmunity in MRL-Fas(lpr) mice (26). To evaluate the temporal effects of CathS inhibition on anti-C1q antibody production, we first used C1q-deficient mice, a model in which EBNA^348-365^ -immunization has been previously shown to induce anti-C1q antibody production (12). C1q-deficient mice received CathS inhibition at different time points following EBNA^348-365^ -immunization: at day 2 (according to the day post-initial immunization), day15 (according to the day post-first booster), or day 30 (according to the day post-second booster), respectively, following a standardized peptide immunization schedule (**Supplementary Figure 2A**). ELISA analyses revealed that CathS inhibition starting from day 0 completely suppressed the generation of anti-EBNA^348-365^, anti-A08, and anti-C1q antibodies, respectively, suggesting a critical role for CathS in the initiation of these immune responses (**Supplementary Figures 2C–E**). Treatment initiated on day 15 or day 30 resulted in unchanged antibody titers of anti-EBNA^348–365^ and anti-A08, indicating that CathS activity is particularly important in the early phase of immunization-induced antibody production (**Supplementary Figures 2F, G, I, J**). In contrast, a significant and progressive decline was observed specifically for anti-C1q antibodies when the inhibitor treatment was started after the first boost (**Supplementary Figure 2H**) while CathS treatment administered after the second boost did not impact anti-C1q antibody titers anymore (**Supplementary Figure 2K**). Total serum IgG concentrations did not differ between CathS-treated and control mice, indicating that CathS inhibition did not affect global IgG production but selectively modulated antigen-specific antibody responses (**Supplementary Figure 2B**). Taken together, these data suggest that antibody formation against the initial immunogen i.e EBNA^348-365^, is antigen presentation-dependent and can be blocked by a CathS inhibitor. In addition, the development of anti-C1q can be partially reduced by CathS inhibitor treatment when applied in a specific time window without affecting anti-EBNA^348–365^ and anti-A08 antibody titers respectively, suggesting that the formation of anti-C1q is an at least partially independent process.

**Figure 2:**
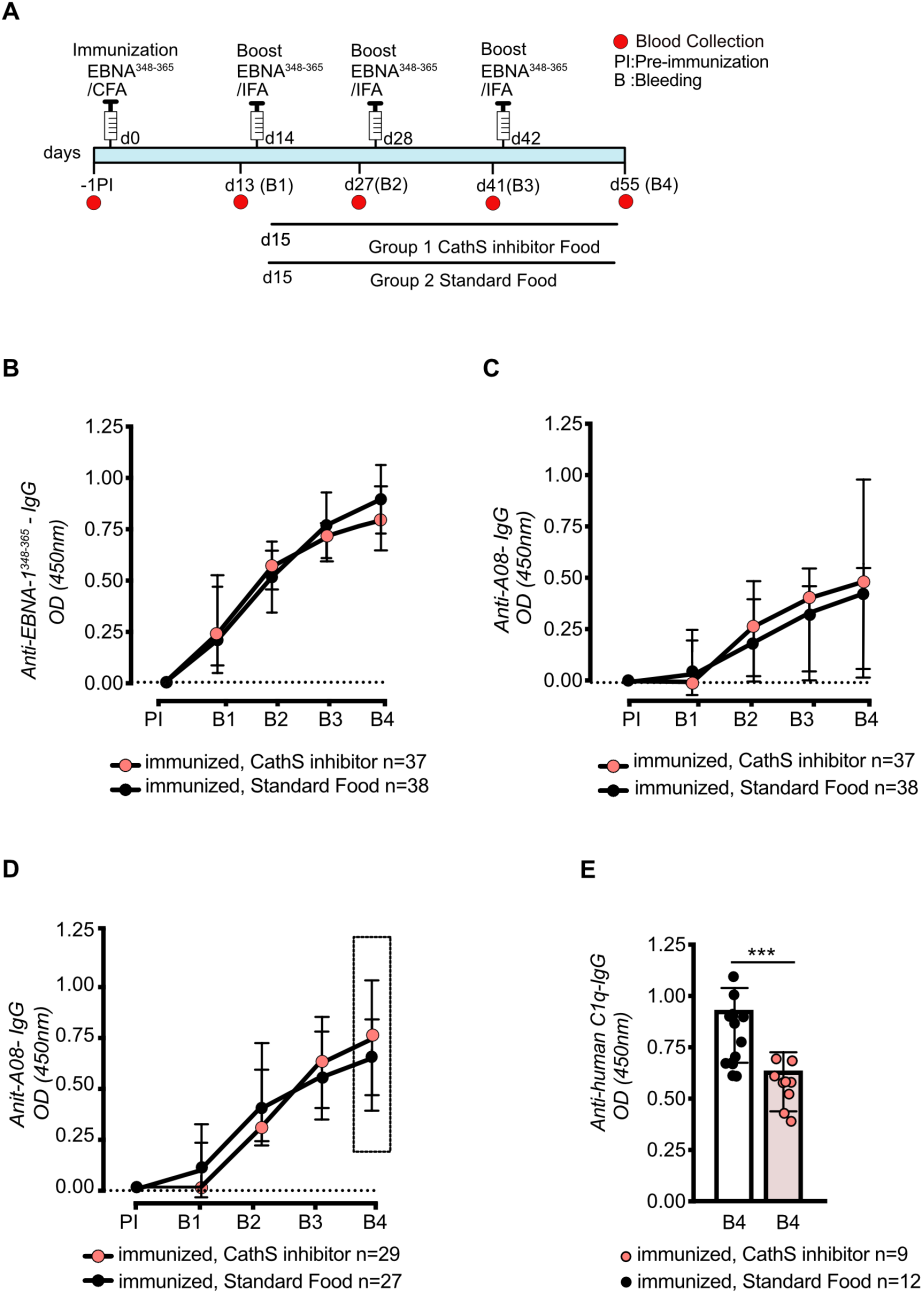
Cathepsin S (CathS) inhibition reduces anti-C1q IgG levels in *Mertk-/-* mice. Experimental design showing the administration of CathS inhibitor-containing diet (Group 1) or standard food (Group 2) after the first boost. (d15). All antibody measurements were performed by ELISA **(A)**. Anti-EBNA^348–365^ IgG levels increased over time in a similar level and in a similar proportion of mice in both groups (CathS inhibitor diet n=37/45; standard food n=38/45) **(B)**. Anti-A08 IgG titers in all immunized mice, including anti-EBNA^348–365^ non-responders, were not significantly different between CathS inhibitor treated and standard-food groups at time point B4 **(C)**. This was also the case, when the analysis was restricted to EBNA^348-365^ -seropositive mice (CathS inhibitor diet n=29/45; standard food n=27/45) **(D)**. However, CathS inhibitor treated mice showed reduced levels of anti-human C1q antibodies at B4, indicating an inhibitory effect on the development of anti- C1q reactivity **(E)**. Data were presented as median with IQR. **(B–D)** or mean ± SD **(E)**. Significant differences were determined using two-tailed unpaired t-test ***P < 0.001.

Having defined a critical treatment window for CathS inhibition, we then administered the compound to *Mertk*-deficient mice (**Figure 2A**). As seen in C1q-deficient mice, the CathS blockade had no effect on anti-EBNA^348–365^ IgG and anti-A08 IgG titers in 37 out of 45 EBNA^348-365^-immunized mice responding to immunization (**Figures 2B, C**). In the standard diet group, 7 out of 45 mice did not mount a detectable anti-EBNA^348–365^ IgG response, while in the CathS-treated group, 8 out of 45 mice failed to develop anti-EBNA^348–365^ IgG following immunization. Anti-A08 IgG levels were analyzed in all mice having developed anti-EBNA^348-365^ (**Figure 2C**) or only in those developing a measurable anti-A08 titer (**Figure 2D**). Among the EBNA^348–365^ responders, 29 out of 37 mice in the CathS-treated group and 27 out of 38 mice in the standard diet group developed anti-A08 IgG titers. Both datasets revealed no statistically significant difference in anti-A08 IgG responses between the groups. With regard to anti-C1q, the number of mice developing the antibody was numerically lower in the CathS-treated group compared to the standard diet, but this difference was not significant (n = 9/29 of anti-EBNA ^348-365^-IgG and anti-A08-IgG double positive mice in the CathS inhibition group developed anti-C1q versus n = 12/27 of anti- EBNA ^348-365^ IgG and anti-A08-IgG double positive mice receiving standard diet) (**Figure 2E**). However, there was a significant reduction in anti-C1q titers in mice having received CathS inhibition compared to the control group, similar to the results seen in C1q-deficient mice (**Figure 2E**; **Supplementary Figure 2H**).

### 3.3 The presence of anti-C1q antibodies is associated with impaired renal function

**Figure 3:**
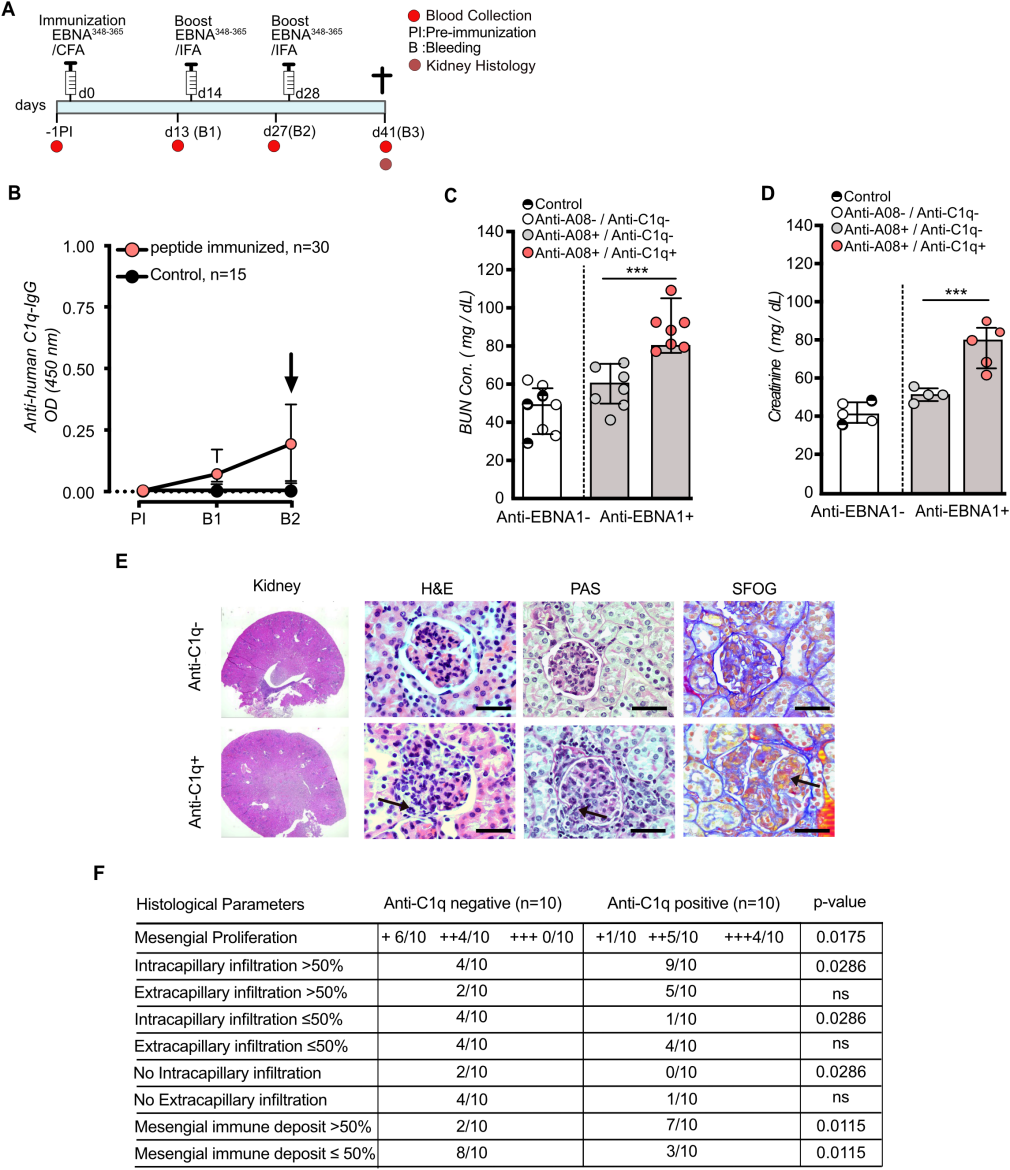
The presence of anti-C1q antibodies is associated with impaired renal function. 7–8 week-old female *Mertk-/-* mice were immunized with EBNA^348–365^ peptide on day 0 and boosted on days 14 and 28. Kidneys were harvested two weeks after the second boost (day 41) for analysis **(A)**. ELISA analyses of serum at B2 (day 27) showed elevated anti-human C1q IgG levels in mice being double positive for anti-A08 and anti-EBNA^348–365^ IgG. Only EBNA^348–365^ responder mice (n = 30) were selected for analysis **(B)**. Blood urea nitrogen (BUN) and serum creatinine concentrations were measured at B3 (day 41) using commercialy available ELISA kits. Due to sample limitations, only 5–7 mice per group were included. Control animals consisted of pooled CFA/IFA-only mice and EBNA^348365^ -immunized non-responders. Comparisons were made between double-positive mice (antiA08^+^/anti-EBNA^348-365 +^) and triple-positive mice (anti-A08^+^/anti-EBNA^348-365 +^/anti-C1q^+^), revealing enhanced renal dysfunction in the triple-positive group **(C, D)**. Representative H&E and PAS-stained kidney sections from EBNA^348-365^ -immunized mice according to anti-C1q IgG status, showing glomerular alterations in anti-C1q positive mice. Arrows indicates increased mesangial cellularity, indicating of mesangial proliferation (H&E), increased inflammatory infiltrates (PAS), and increased immune complex deposits and extracellular matrix expansion (Sfog) **(E)**. Histological scoring showed significantly increased mesangial proliferation intracapillary infiltration, and mesangial immune deposits in anti-C1q positive mice (n=10 per group) **(F)**. Data were presented as median with IQR **(B)** and mean ± SD **(C, D)**, respectively. Statistical significance was determined using two-tailed Mann– Whitney U test **(B)** and Kruskal-Wallis test **(C, D)**. Mesangial proliferation and all other histological parameters were evaluated using a one-tailed Fisher’s exact test **(F)**. **P < 0.01, ***P < 0.001, ***P < 0.001; ns, not significant.

In SLE, the presence of anti-C1q antibodies is typically associated with proliferative lupus nephritis. Therefore, we performed a separate set of experiments with focus on kidney histology (**Figure 3A**). Anti-C1q titers in the lupus model analyzed here began to increase significantly after the second boost (d27) (**Figure 3B**). Therefore, we next analyzed the renal function based on Blood Urea Nitrogen (BUN) and creatinine at this timepoint. Three groups of mice were compared: The control group included both, adjuvant-only mice and EBNA^348-365^-immunized mice that did not develop a detectable anti-EBNA^348–365^ IgG response. The second group comprised mice being double positive (i.e. anti-EBNA+/anti-/A08+), but did not develop anti-C1q antibodies. The third group was triple positive (anti-EBNA+/anti-/A08+, anti-C1q+). BUN and creatinine levels were significantly higher in the triple positive group, while the sera from double positive mice lacking anti-C1q antibodies showed no significant difference compared to the pooled controls (p < 0.001, Kurskal-Wallis test, respectively **Figures 3C, D**). To elucidate the histopathological basis underlying the renal dysfunction, we performed light microscopy using various staining techniques, including Hematoxylin and eosin (H&E), Periodic Acid-Schiff (PAS), and silver staining (sFOG). Comparing the triple-positive mice (i.e. anti-C1q positive) with the corresponding double-positive but anti-C1q negative group, we observed a significant increase in mesangial proliferation with varying severity (p = 0.0125). Regarding subgroups, anti-C1q–positive mice were numerically more diseased across all categories analyzed, and all anti-C1q–positive mice developed more intra- and extracapillary infiltration; however, differences were not significant for extracapillary infiltration. We then analysed anti-C1q–positive and anti-C1q–negative mice by categorizing responses as “50 percent or less” versus “more than 50 percent”. A one-tailed Fisher test revealed a significant difference in response distribution between the two groups (p = 0.0286), indicating that anti-C1q status is associated with distinct patterns of glomerular immune infiltration.

**Figure 4:**
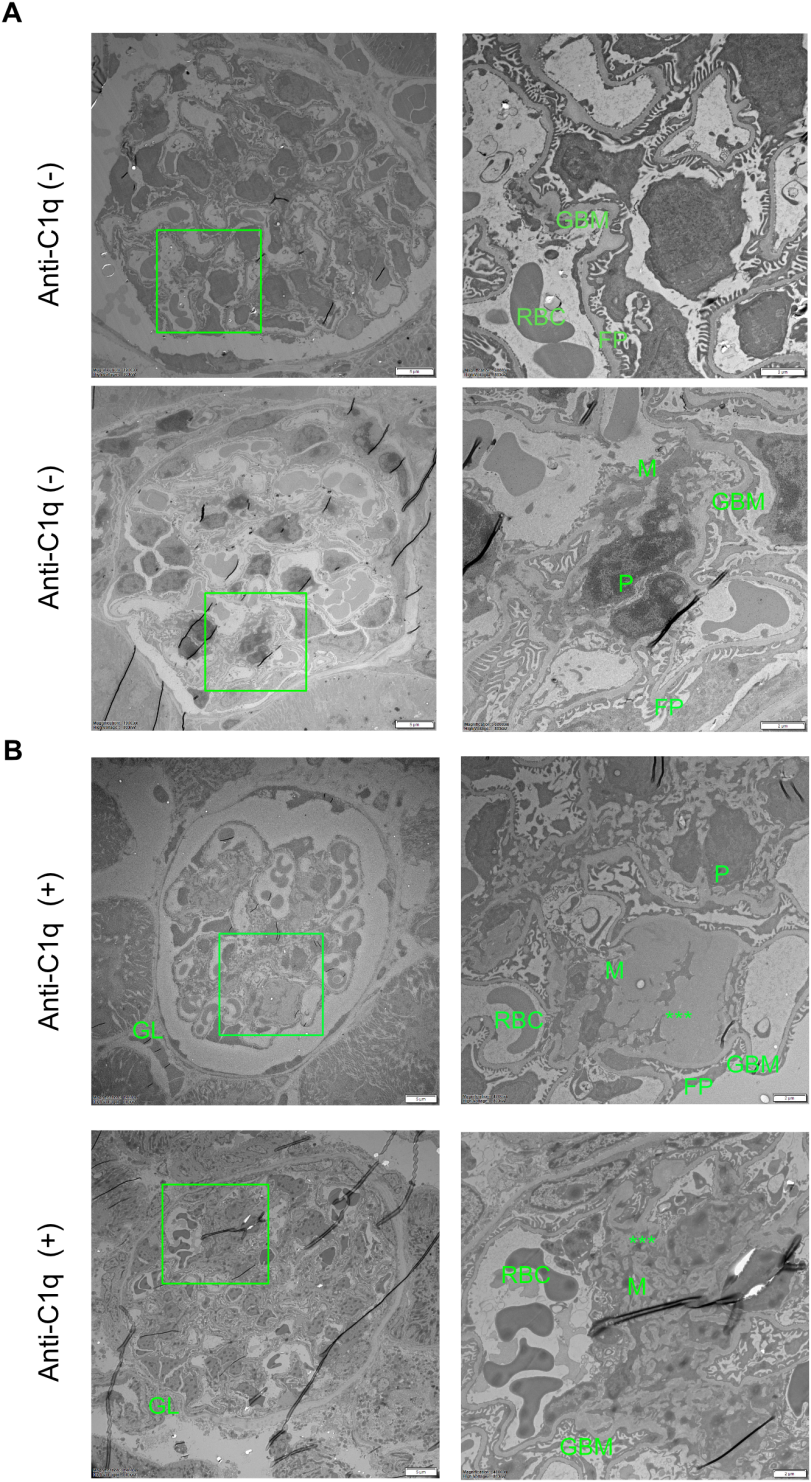
Increased glomerular immune complex deposition is associated with anti-C1q status. Representative electron microscopy images of mesangial deposits in kidneys from anti-C1q–negative and anti-C1q–positive mice. GBM: glomerular basement membrane; P: podocyte; M: mesangial area; RBC: red blood cell; FP: foot processes. Green brackets indicate the regions where deposits are focused. Asterisks (***) mark electron-dense immune deposits. Left panel: scale bar, 5 μm; right panel: scale bar, 2 μm. **(A, B)**.

Overall, the increased mesangial proliferation observed in triple-positive mice (anti-EBNA^348–365^, anti-A08, and anti-C1q) is consistent with an association between anti-C1q positivity and enhanced glomerular pathology. Representative glomerular images are shown in **Supplementary Figure 3** (n = 3 mice per group), whereas categorical histopathological scoring across the full cohort (reported as n/N per category) and the corresponding proportion-based statistical analyses are presented in **Figure 3F** (see also **Supplementary Figure 3**). We further analyzed the kidney sections using electron microscopy to assess structural differences between anti-C1q positive and anti-C1q negative groups. The analyses revealed increased immune complex deposition accompanied by mesangial expansion, i.e. mesangial proliferative glomerulonephritis, in anti-C1q positive mice (**Figures 4A, B**).

### 3.4 IgG-deposition and complement-protein-deposition are increased in anti-C1q positive compared to anti-C1q negative mice

**Figure 5:**
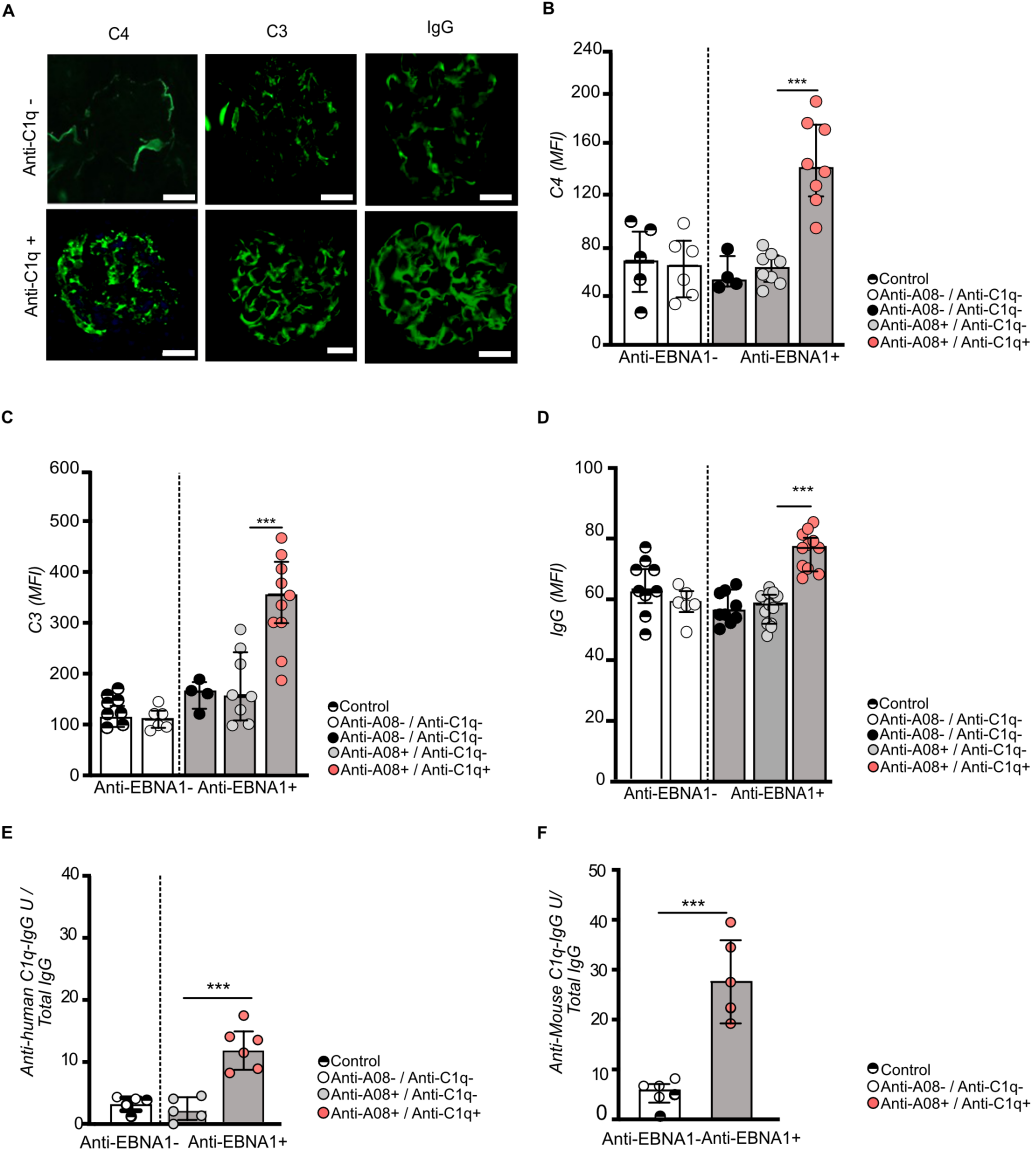
Anti-C1q positivity is associated with increased glomerular deposition of complement and IgG. Quantification of glomerular C3, C4, and total IgG deposition by mean fluorescence intensity (MFI), assessed by immunofluorescence microscopy in kidney sections from EBNA^348–365^ responder mice stratified by serostatus into anti-A08^+^/anti-C1q⁻ and anti-A08^+^/anti-C1q^+^ groups, as well as negative controls **(A–D)**. Anti-C1q antibody reactivity in kidney eluates from EBNA^348–365^ responder mice. IgGs were eluted from kidney tissue of pooled control mice (adjuvanttreated and EBNA^348–365^ non-responders), double-positive (anti-A08^+^/anti-C1q⁻), and triple-positive (anti-A08^+^/anti-C1q^+^) mice. Eluted anti-C1q antibodies from triple-positive mice bound both human **(E)** and mouse C1q **(F)**. Data are shown as mean ± SEM. Statistical comparisons used ANOVA or unpaired t-tests; *P < 0.05, **P < 0.01, ***P < 0.001.

Complement proteins C3 and C4 are down-stream complement components of C1q, playing a central role in the clearance of immune complexes and the regulation of inflammatory responses. In lupus nephritis, dysregulated complement activation contributes to tissue damage, making C3 and C4 key biomarkers for disease progression. C3 and C4 consumption is seen in serum associated with SLE flares, while deposition in kidney biopsies is used as marker for lupus nephritis. Therefore, we next analysed complement- and IgG-deposition in kidney sections using immunofluorescence in EBNA^348-365^-immunized and non-immunized mice. Mice positive for anti-C1q exhibited significantly higher levels of C4, C3 and IgG deposition compared to anti-C1q-negative mice, indicating enhanced complement activation in the presence of this autoantibody (**Figures 5A–D**). Moreover, complement as well as IgG deposition was only significantly higher in mice positive for both, anti-C1q and anti-A08, indicating that the presence of antibodies recognizing intact C1q was essential for the enhancement of immune deposits (21).

Last, to demonstrate that anti-C1q autoantibodies were deposited in the diseased kidney as shown for patients with lupus nephritis, we eluted immunoglobulins from kidneys comparing pooled samples from control mice (adjuvant-treated and EBNA-immunized non-responders), double positive mice (Anti-EBNA ^358-365^, Anti-A08) and triple positive mice (Anti-EBNA ^358-365^, Anti-A08, Anti-C1q). Notably, anti-C1q reactivity of the kidney eluate was exclusively detected in eluates from mice with positive serum-antibody titers for both anti-A08 and anti-C1q antibodies (**Figure 5E**). As already shown for plasma antibody levels, eluated anti-C1q antibodies recognized both humanC1q as well as mouse C1q (**Figure 5F**).

## 4 Discussion

This study provides *in vivo* evidence that an EBV–derived peptide can trigger the production of pathogenic autoantibodies against complement C1q (anti-C1q) in lupus-prone mice. These induced anti-C1q share characteristics with anti-C1q seen in SLE patients, i.e. being high affinity IgG autoantibodies targeting the collagen-like region of C1q (27). Furthermore, the generation of anti-C1q is a specific result of EBNA-1 peptide immunization, as we did not detect anti-C1q in non-immunized or control-peptide immunized mice, and we did not observe enhanced levels of anti-dsDNA in parallel. In addition, the induced anti-C1q appear to be pathogenic as an accelerated glomerulonephritis was only observed in anti-C1q positive mice but not in anti-C1q negative ones that underwent the same immunization procedure, and anti-C1q could only be eluted from the renal cortex of anti-C1q positive mice.

In our experimental setup the EBV-derived peptide was applied to *Mertk*-deficient mice that have a genetically determined defect in the clearance of apoptotic cells. This background is also relevant to renal disease, as loss of *Mertk* worsens immune-mediated nephritis in nephrotoxic serum models, consistent with impaired efferocytosis and dysregulated inflammatory environment sensitizing the kidney to immune-complex/complement-driven injury (28, 29). This allowed us to explore a combination of factors that are considered to play an important role in the development of human SLE. Namely, we combined the hampered clearance of apoptotic cells as a genetic predisposition with exposure to an environmental trigger such as EBV infection. In this context it is of interest to note, that human anti-C1q autoantibodies had been described to specifically recognise C1q being bound to apoptotic cells (30). The genetic background of *Mertk* mice leading to an altered clearance of apoptotic cells allowed the induction of anti-C1q autoantibodies resembling those that we previously observed in C1q-deficient mice, not having developed tolerance for C1q. The fact that induction of anti-C1q in wildtype mice was not possible using a similar immunization protocol in a previous study (12) suggests that a defective clearance of apoptotic cells alone allows the required break of tolerance against C1q.

C1q deficiency as a genetic risk factor for SLE and C1q deposition in lupus nephritis describe different contexts. Inherited absence of C1q is believed to promote lupus by altering the clearance of apoptotic debris/immune complexes (31) and weakening tolerance, thereby facilitating autoantibody development. Conversely, in C1q-sufficient disease, C1q deposits in glomeruli together with immune complexes and can locally promote classical pathway activation, providing a substrate for anti-C1q–mediated amplification of renal inflammation (32).

Anti-C1q as induced in our model of SLE seem to be the result of a two-step process: in parallel to the development of anti- EBNA^348–365^ antibodies we observed a rise in anti-A08 antibodies that initially did not or only partially recognise the intact C1q molecule, too. The occurrence of anti-C1q seem to be a secondary phenomenon, while the generation of anti-A08 seem to be a prerequisite for the development of anti-C1q, as anti-C1q were only detected in anti-A08 positive mice. This observation supports the hypothesis that anti-C1q were induced by molecular mimicry and consequent epitope spreading. This supposed epitope spreading could be partially prevented by a drug intervention, i.e. inhibition of CathS underlining not only the delayed character of anti-C1q formation but also supporting the hypothesis of a dependency on antigen presentation. However, as CathS inhibition was shown to also influence additional inflammatory pathway (26) our findings cannot be considered as a proof of a T cell dependent process. Interestingly, anti-A08 not binding complete C1q seem not to be pathogenic but may represent an intermediate step only. Thus, our experiments delineate a certain hierarchy of mimicry after exposure to the EBV-antigen, with three immunologic states: (i) no cross-reactivity, (ii) anti-A08 reactivity without C1q recognition, and (iii) cross-reactivity with intact C1q. Only the latter was found to be associated with renal dysfunction, suggesting that only the recognition of full C1q is able to mediate tissue injury. Although formally not proven, the transition from anti-A08-limited to full anti-C1q reactivity in our model most likely reflects B cell–T cell cooperation and antigen presentation, marking a potential checkpoint in disease acceleration, that offers the possibility of a drug intervention, e.g. early treatment intervention after primary EBV infection in susceptible individuals.

Furthermore, not all immunized mice developed anti-C1q autoantibodies. This may be well explained by both technical and biological variation. Minor differences in immunogen preparation, administration, or housing could significantly affect the initial response. In addition, immunised lupus-prone mice may generate heterogeneous memory B cell pools as observed e.g. after SARS-CoV-2 vaccination (33, 34).

Together with the growing evidence for a link between EBV infection and multiple sclerosis through molecular mimicry—particularly the homology between EBNA1 and the CNS protein GlialCAM—our data also highlight the need for preventive strategies targeting the earliest stages of autoimmune initiation (35). Developing vaccines that prevent or attenuate EBV infection—especially by neutralizing key viral antigens like EBNA1—could disrupt the pathogenic loop before it begins, offering a promising avenue for autoimmune disease prevention in genetically predisposed individuals.

Our study adds to the growing body of evidence implicating EBV epitopes in the pathogenesis of SLE through molecular mimicry (36, 37). While Tu et al. (2018) identified ten linear B-cell epitopes from EBV early antigens, membrane antigens, and latent membrane proteins (LMP-1 and LMP-2A) that share moderate to high sequence and structure similarity with canonical lupus autoantigens (e.g., SmB, SmD, Ro, rRNP), our study provides now direct functional evidence for a distinct and pathogenic form of mimicry (38). Unlike Tu et al.’s epitopes—which primarily mimic nuclear autoantigens and promote ANA production— we demonstrate that the EBNA^348–365^ peptide elicits cross-reactive antibodies that can recognize native C1q based on sequence identity. This interaction is independent of gene regulatory or epigenetic mechanisms described for other EBV proteins such as EBNA2 and EBNA3C, and instead reflects a direct, B-cell receptor (BCR)-driven break in tolerance. Thus, EBNA^348-365^/A08 of C1q expands the repertoire of EBV-host epitope mimicry beyond spliceosomal antigens, highlighting the role of viral peptides in shaping autoreactivity in lupus.

Clinical findings further contextualize our results. Chen et al. (2024) reported that EBV-VCA IgA and EBV-EA IgG levels—markers of viral reactivation—are significantly elevated in patients with active SLE. These data suggest a feed-forward loop in which EBV reactivation fuels recurrent antigenic stimulation and autoimmune priming (39). Recent work by Goetzke et al. (2025) demonstrated that TGFβ blockade restores EBV-specific cytotoxic T-cell responses in the setting of hyperinflammatory signalling (40). Although the role of TGFβ in SLE remains incompletely understood, these findings suggest that targeting the impaired T cell–mediated control of EBV may represent another promising therapeutic strategy in autoimmune settings such as SLE, where viral latency and immune dysregulation are intertwined.

As a limitation, our study does not allow to clearly define down-stream actions of the induced anti-C1q causing the observed acceleration of kidney disease. However, our data would be well in line with a previous study on monoclonal anti-C1q by Trouw et al. showing that monoclonal anti-C1q mediate their pathogenic effect via complement activation and Fc receptors (32). Mesangial proliferation is a hallmark of early lupus nephritis and reflects the deposition of immune complexes within the glomerular mesangium. The additional presence of anti-C1q autoantibodies might initiate a cascade of pro-inflammatory events that recruit immune cells, activate mesangial cells, and trigger expansion of the extracellular matrix. This cellular proliferation is not merely a histological feature but a pathogenic driver of glomerular injury. Because mesangial proliferation can become irreversible if not controlled early, the prevention of this early inflammatory amplification is critical to preserve the renal architecture and to avoid progression towards chronic kidney damage.

Another limitation of our study is the use of an EBV-derived peptide rather than full viral EBV infection. As EBV is a human-specific pathogen, direct modeling of EBV infection in mice is not straightforward. Moreover, EBV infection or the use of complete EBNA-1 protein as an immunogen are expected to trigger a broad spectrum of autoreactive responses, which would obscure the specific contribution of anti-C1q autoantibodies to the observed pathogenic effects. Last, while our data on CathS inhibition support a checkpoint role at the level of antigen processing and T cell–B cell cooperation, CathS also regulates inflammatory mediators, antimicrobial defense, and tissue homeostasis. Accordingly, CathS inhibition would be expected to *attenuate* rather than completely abrogate antigen-specific humoral responses *in vivo*, because antigen presentation can be partially maintained through redundant endolysosomal proteases and alternative processing routes (25, 41, 42). In line with this, CatS inhibition is unlikely to fully block MHC class II antigen processing, as other proteases (e.g., cathepsins L and B and asparagine endopeptidase) can partially compensate and sustain peptide generation. In addition, once class-switched responses are established, circulating IgG can remain readily detectable due to pre-existing antibody pools and the relatively long serum half-life of IgG; thus, reduced *de novo* priming would be expected to dampen, but not eliminate, ELISA signals. Therefore, future studies using cell-specific or pathway-specific targeting will be needed to disentangle the relative contributions of these mechanisms. In addition, future immunophenotyping of immune cell subsets before and after EBNA^348–365^ immunization would provide deeper insight into the specific B- and T-cell populations contributing to anti-C1q induction and disease exacerbation.

In conclusion, our study demonstrates that the EBV-derived peptide EBNA^348–365^ can induce pathogenic anti-C1q autoantibodies through molecular mimicry in a lupus-prone mouse model. The epitope spreading from non-pathogenic anti-A08-antibodies to kidney-disease accelerating anti-C1q antibodies was found to be at least in part dependent on antigen presentation and could be attenuated by the application of a CathS Inhibitor. These findings not only validate a mechanistic link between EBV, anti-C1q and proliferative lupus nephritis but also highlight early immunologic checkpoints and viral targets as promising avenues for disease prevention and intervention.

## Data Availability

The original contributions presented in the study are included in the article/**Supplementary Material**. Further inquiries can be directed to the corresponding author.

## Supplementary material

Supplementary Figure 1 Classification of autoantibody responses following EBNA348 immunization. ELISA quantification of serum anti-EBNA^348–365^ IgG levels in peptide -immunized mice (n=45) versus adjuvant-only controls (n=15) at indicated time points (PI, B1–B4) **(A)**. Anti-A08 titers increased significantly over time when including all mice having undergone the immunisation procedure **(B)**. No anti-C1q could be observed in peptide (C08)-control mice **(C)**. Anti-human C1q IgG subclass analysis in peptide-immunized mice. Sera from triple-positive, double-, and non-responder peptide-immunized mice were analyzed by ELISA for total IgG, IgG subclasses (IgG2c/2b, IgG3, IgG1), IgM, and IgA. **(D)** Competition assay demonstrating specific inhibition of anti-C1q antibody binding to human C1q by the A08 peptide but not by the scrA08 peptide. Data are shown as percentage of binding across increasing peptide concentrations. Data are presented as median with IQR **(A–D)**. Data are presented as a line graph showing % binding on the y-axis versus peptide concentration (μg/mL) on a logarithmic x-axis.**(E)** Statistical significance was determined using two-tailed Mann–Whitney U test. **P < 0.01, ***P < 0.001.

Supplementary Figure 2 CathS inhibitor administration attenuates anti-C1q IgG levels in *C1q_a_-/-* mice when administered early, but not at later time points. Immunization schedules and dietary regimen used in three independent cohorts, each randomized to receive standard diet or a CathS inhibitor diet in three different intervention arms **(A)**. Longitudinal analysis of total serum IgG levels in mice immunized with EBNA^348–365^ peptide and fed either CathS inhibitor diet or standard diet starting at day 15 post-immunization (n = 15/group). **(B)** Longitudinal analysis of serum, antiEBNA^348–365^ IgG, anti-A08 IgG, and anti-human C1q IgG in CathS inhibitor diet versus standart diet groups across all cohorts (cohort sizes: n=13–39/group). CathS inhibitor treatment significantly reduced all antibody levels when introduced directly after the start of the immunization schedule **(C–E)**, while there was no effect when introduced late (at day 30) **(I–K)**. In contrast, CathS inhibition significantly reduced anti-C1q levels at B3 and B4 without affecting the primary immunogenicity against EBNA^348–365^ when started at day 15 of the immunization schedule **(F–H)** Statistical comparisons were made using repeated-measures ANOVA or two-tailed unpaired t-tests as appropriate; *P < 0.05, **P < 0.01, ***P < 0.001.

Supplementary Figure 3 Renal histopathology in anti-C1q– and anti-C1q+ mice. Representative glomerular sections stained with H&E, PAS, and Sfog from EBNA^348–365^ immunized mice grouped according to anti-C1q status. Red arrows indicate glomerular abnormalities including mesangial proliferation (H&E), mesangial matrix expansion and increased cell filtration (PAS), and collagen deposition accumulation. (SFOG)Scale bar = 20 μm.

## References

[1] Arbuckle MR McClain MT Rubertone MV Scofield RH Dennis GJ James JA . Development of autoantibodies before the clinical onset of systemic lupus erythematosus. New Engl J Med. (2003) 349:1526–33. doi: 10.1056/nejmoa021933, PMID: 14561795

[2] Kaul A Gordon C Crow MK Touma Z Urowitz MB Van Vollenhoven R . Systemic lupus erythematosus. Nat Rev Dis Primers. (2016) 2:16039. doi: 10.1038/nrdp.2016.39, PMID: 27306639

[3] Murphy G Lisnevskaia L Isenberg D . Systemic lupus erythematosus and other autoimmune rheumatic diseases: Challenges to treatment. Lancet. (2013) 382:S62. doi: 10.1016/S0140-6736(13)60889-2, PMID: 23972423

[4] Pickering MC Walport MJ . Links between complement abnormalities and systemic lupus erythematosus. Rheumatology. (2000) 39:133–41. doi: 10.1093/rheumatology/39.2.133, PMID: 10725062

[5] Botto M Walport MJ . C1q, autoimmunity and apoptosis. Immunobiology. (2002) 205:395–406. doi: 10.1078/0171-2985-00141, PMID: 12396002

[6] Jennette JC Hipp CG . Immunohistopathologic evaluation of C1q in 800 renal biopsy specimens. Am J Clin Pathol. (1985) 83:415–20. doi: 10.1093/ajcp/83.4.415, PMID: 3885712

[7] Trendelenburg M Lopez-Trascasa M Potlukova E Moll S Regenass S Frémeaux-Bacchi V . High prevalence of anti-C1q antibodies in biopsy-proven active lupus nephritis. Nephrol Dialysis Transplant. (2006) 21:3115–21. doi: 10.1093/ndt/gfl436, PMID: 16877491

[8] Reid KBM . Complement component C1q: Historical perspective of a functionally versatile, and structurally unusual, serum protein. Front Immunol. (2018) 9:764. doi: 10.3389/fimmu.2018.00764, PMID: 29692784 PMC5902488

[9] Poole BD Scofield RH Harley JB James JA . Epstein-Barr virus and molecular mimicry in systemic lupus erythematosus. Autoimmunity. (2006) 39:63–70. doi: 10.1080/08916930500484849, PMID: 16455583

[10] Chen M Tu J Huang M Cheng Y Sun L . A retrospective cohort study of Epstein-Barr virus infection status and systemic lupus erythematosus. Clin Rheumatol. (2024) 43:1235–42. doi: 10.1007/s10067-024-06917-4, PMID: 38509241

[11] Bjornevik K Cortese M Healy BC Kuhle J Mina MJ Leng Y . Longitudinal analysis reveals high prevalence of Epstein-Barr virus associated with multiple sclerosis. Sci (1979). (2022) 375:296–301. doi: 10.1126/science.abj8222, PMID: 35025605

[12] Csorba K Schirmbeck LA Tuncer E Ribi C Roux-Lombard P Chizzolini C . Anti-C1q antibodies as occurring in systemic lupus erythematosus could be induced by an epstein-barr virus-derived antigenic site. Front Immunol. (2019) 10:2619. doi: 10.3389/fimmu.2019.02619, PMID: 31787984 PMC6853867

[13] Laurynenka V Ding L Kaufman KM James JA Harley JB . A high prevalence of anti-EBNA1 heteroantibodies in systemic lupus erythematosus (SLE) supports anti-EBNA1 as an origin for SLE autoantibodies. Front Immunol. (2022) 13:830993. doi: 10.3389/fimmu.2022.830993, PMID: 35251022 PMC8892314

[14] Munroe ME Anderson JR Gross TF Stunz LL Bishop GA James JA . Epstein-barr functional mimicry: pathogenicity of oncogenic latent membrane protein-1 in systemic lupus erythematosus and autoimmunity. Front Immunol. (2021) 11:606936. doi: 10.3389/fimmu.2020.606936, PMID: 33613527 PMC7886997

[15] Laurynenka V Ding L Kottyan LC Weirauch MT Kaufman KM James JA . 1706 A model of lupus pathogenesis: anti-EBNA1 heteroantibodies initiate lupus by cross reacting with lupus autoantigens, resulting in lupus autoantibodies and clinical disease. Lupus Sci Med. (2021) 8:A68–9. doi: 10.1136/lupus-2021-lupus21century.101, PMID: 41802225

[16] McClain MT Heinlen LD Dennis GJ Roebuck J Harley JB James JA . Early events in lupus humoral autoimmunity suggest initiation through molecular mimicry. Nat Med. (2005) 11:85–9. doi: 10.1038/nm1167, PMID: 15619631

[17] Yadav P Tran H Ebegbe R Gottlieb P Wei H Lewis RH . Antibodies elicited in response to EBNA-1 may cross- react with dsDNA. PLoS One. (2011) 6:e14488. doi: 10.1371/journal.pone.0014488, PMID: 21245919 PMC3014975

[18] Maguire C Wang C Ramasamy A Fonken C Morse B Lopez N . Molecular mimicry as a mechanism of viral immune evasion and autoimmunity. Nat Commun. (2024) 15:9403. doi: 10.1038/s41467-024-53658-8, PMID: 39477943 PMC11526117

[19] Fejer G Koroknai A Banati F Györy I Salamon D Wolf H . Latency type-specific distribution of epigenetic marks at the alternative promoters Cp and Qp of epstein-barr virus. J Gen Virol. (2008) 89:2260–70. doi: 10.1099/vir.0.83594-0, PMID: 18474551

[20] McClain MT Poole BD Bruner BF Kaufman KM Harley JB James JA . An altered immune response to Epstein-Barr nuclear antigen 1 in pediatric systemic lupus erythematosus. Arthritis Rheum. (2006) 54:360–8. doi: 10.1002/art.21682, PMID: 16385527

[21] Kleer JS Rabatscher PA Weiss J Leonardi J Vogt SB Kieninger-Gräfitsch A . Epitope-specific anti-C1q autoantibodies in systemic lupus erythematosus. Front Immunol. (2022) 12:761395. doi: 10.3389/fimmu.2021.761395, PMID: 35087514 PMC8788646

[22] Cohen PL Caricchio R Abraham V Camenisch TD Charles Jennette J Roubey RAS . Delayed apoptotic cell clearance and lupus-like autoimmunity in mice lacking the c-mer membrane tyrosine kinase. J Exp Med. (2002) 196:135–40. doi: 10.1084/jem.20012094, PMID: 12093878 PMC2194017

[23] Schots A van der Leede BJ De Jongh E Egberts E . A method for the determination of antibody affinity using a direct ELISA. J Immunol Methods. (1988) 109:155–9. doi: 10.1016/0022-1759(88)90247-5, PMID: 3361133

[24] Wener MH Uwatoko S Mannik M . Antibodies to the collagen-like region of C1q in sera of patients with autoimmune rheumatic diseases. Arthritis Rheum. (1989) 32:544–51. doi: 10.1002/anr.1780320506, PMID: 2785797

[25] Riese RJ Mitchell RN Villadangos JA Shi GP Palmer JT Karp ER . Cathepsin S activity regulates antigen presentation and immunity. J Clin Invest. (1998) 101:2351–63. doi: 10.1172/JCI1158, PMID: 9616206 PMC508824

[26] Tato M Kumar SV Liu Y Mulay SR Moll S Popper B . Cathepsin S inhibition combines control of systemic and peripheral pathomechanisms of autoimmune tissue injury. Sci Rep. (2017) 7:1894. doi: 10.1038/s41598-017-01894-y, PMID: 28584258 PMC5459853

[27] Trendelenburg M . Autoantibodies against complement component C1q in systemic lupus erythematosus. Clin Transl Immunol. (2021) 10:e1279. doi: 10.1002/cti2.1279, PMID: 33968409 PMC8082710

[28] Shao WH Zhen Y Rosenbaum J Eisenberg RA McGaha TL Birkenbach M . A protective role of Mer receptor tyrosine kinase in nephrotoxic serum-induced nephritis. Clin Immunol. (2010) 136:224–31. doi: 10.1016/j.clim.2010.04.002, PMID: 20444650 PMC2902650

[29] Zhen Y Priest SO Shao W-H . Opposing roles of tyrosine kinase receptors mer and axl determine clinical outcomes in experimental immune-mediated nephritis. J Immunol. (2016) 197:187–94. doi: 10.4049/jimmunol.1600793, PMID: 27527599 PMC5010948

[30] Bigler C Schaller M Perahud I Osthoff M Trendelenburg M . Autoantibodies against complement C1q specifically target C1q bound on early apoptotic cells. J Immunol. (2009) 182:6608–16. doi: 10.4049/jimmunol.0803573, PMID: 19648280

[31] Botto M Dell’Agnola C Bygrave AE Thompson EM Cook HT Petry F . Homozygous C1q deficiency causes glomerulonephritis associated with multiple apoptotic bodies. Nat Genet. (1998) 19:56–9. doi: 10.1038/ng0598-56, PMID: 9590289

[32] Trouw LA Groeneveld TWL Seelen MA Duijs JMGJ Bajema IM Prins FA . Anti C1q autoantibodies deposit in glomeruli but are only pathogenic in combination with glomerular C1q-containing immune complexes. J Clin Invest. (2004) 114:1057–66. doi: 10.1172/JCI200421075, PMID: 15343386 PMC514584

[33] He B Liu S Xu M Hu Y Lv K Wang Y . Comparative global B cell receptor repertoire difference induced by SARS-CoV-2 infection or vaccination via single-cell V(D)J sequencing. Emerg Microbes Infect. (2022) 11:2041–56. doi: 10.1080/22221751.2022.2105261, PMID: 35899581 PMC9377262

[34] Kotagiri P Mescia F Rae WM Bergamaschi L Tuong ZK Turner L . B cell receptor repertoire kinetics after SARS-CoV-2 infection and vaccination. Cell Rep. (2022) 38:110393. doi: 10.1016/j.celrep.2022.110393, PMID: 35143756 PMC8801326

[35] Lanz TV Brewer RC Ho PP Moon JS Jude KM Fernandez D . Clonally expanded B cells in multiple sclerosis bind EBV EBNA1 and GlialCAM. Nature. (2022) 603:321–7. doi: 10.1038/s41586-022-04432-7, PMID: 35073561 PMC9382663

[36] Poole BD Gross T Maier S Harley JB James JA . Lupus-like autoantibody development in rabbits and mice after immunization with EBNA-1 fragments. J Autoimmun. (2008) 31:362–71. doi: 10.1016/j.jaut.2008.08.007, PMID: 18849143 PMC2852321

[37] Harley JB Clark DH Chepelev I . 101 Anti-EBNA1 molecular mimicry initiates cross-reacting autoantibodies in Systemic Lupus Erythematosus (SLE) & Multiple Sclerosis (MS). Lupus Sci Med. (2022) 9:A1. doi: 10.1136/lupus-2022-lupus21century.1, PMID: 41802225

[38] Tu J Wang X Geng G Xue X Lin X Zhu X . The possible effect of B-cell epitopes of Epstein-Barr virus early antigen, membrane antigen, latent membrane protein-1, and -2A on systemic lupus erythematosus. Front Immunol. (2018) 9:187. doi: 10.3389/fimmu.2018.00187, PMID: 29497417 PMC5819577

[39] Chen CJ Lin KH Lin SC Tsai WC Yen JH Chang SJ . High prevalence of immunoglobulin A antibody against Epstein-Barr virus capsid antigen in adult patients with lupus with disease flare: Case control studies. J Rheumatol. (2005) 32:1662–6. 15630723

[40] Goetzke CC Massoud M Frischbutter S Guerra GM Ferreira-Gomes M Heinrich F . TGFβ links EBV to multisystem inflammatory syndrome in children. Nature. (2025) 639:198–206. doi: 10.1038/s41586-025-08697-6, PMID: 40074901 PMC12003184

[41] Shen L Sigal LJ Boes M Rock KL . In this process, exogenous antigens are internalized into endocytic compartments. J Immunol. (2004) 173:5672–8. doi: 10.4049/jimmunol.173.9.5672, PMID: 34749531

[42] Allan ERO Yates RM . Redundancy between cysteine cathepsins in murine experimental autoimmune encephalomyelitis. PLoS One. (2015) 10:e0128945. doi: 10.1371/journal.pone.0128945, PMID: 26075905 PMC4468166

