# Supplementary figures and images for: Epstein Barr virus antigen-induced autoantibodies against complement C1q exacerbate renal disease in lupus-prone mice
Source: Front Immunol. 2026 Mar 18;17:1710424. doi: 10.3389/fimmu.2026.1710424 (PMC13039013; doi:10.3389/fimmu.2026.1710424)

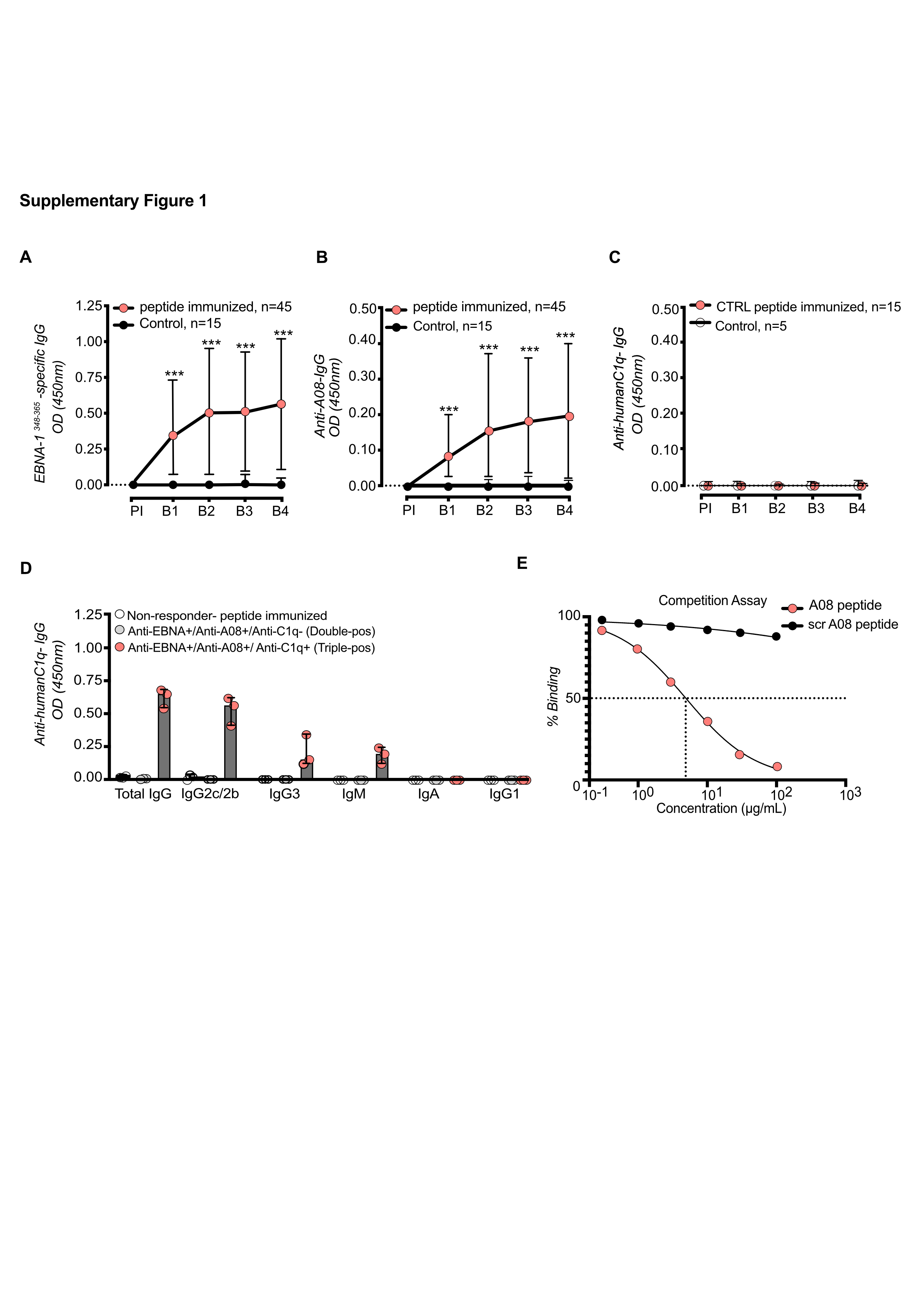

Supplement: Supplementary Figure 1 — Classification of autoantibody responses following EBNA348 immunization. ELISA quantification of serum anti-EBNA348–365 IgG levels in peptide -immunized mice (n=45) versus adjuvant-only controls (n=15) at indicated time points (PI, B1–B4) (A). Anti-A08 titers increased significantly over time when including all mice having undergone the immunisation procedure (B). No anti-C1q could be observed in peptide (C08)-control mice (C). Anti-human C1q IgG subclass analysis in peptide-immunized mice. Sera from triple-positive, double-, and non-responder peptide-immunized mice were analyzed by ELISA for total IgG, IgG subclasses (IgG2c/2b, IgG3, IgG1), IgM, and IgA. (D) Competition assay demonstrating specific inhibition of anti-C1q antibody binding to human C1q by the A08 peptide but not by the scrA08 peptide. Data are shown as percentage of binding across increasing peptide concentrations. Data are presented as median with IQR (A–D). Data are presented as a line graph showing % binding on the y-axis versus peptide concentration (μg/mL) on a logarithmic x-axis.(E) Statistical significance was determined using two-tailed Mann–Whitney U test. **P < 0.01, ***P < 0.001. [file Image1.tiff]

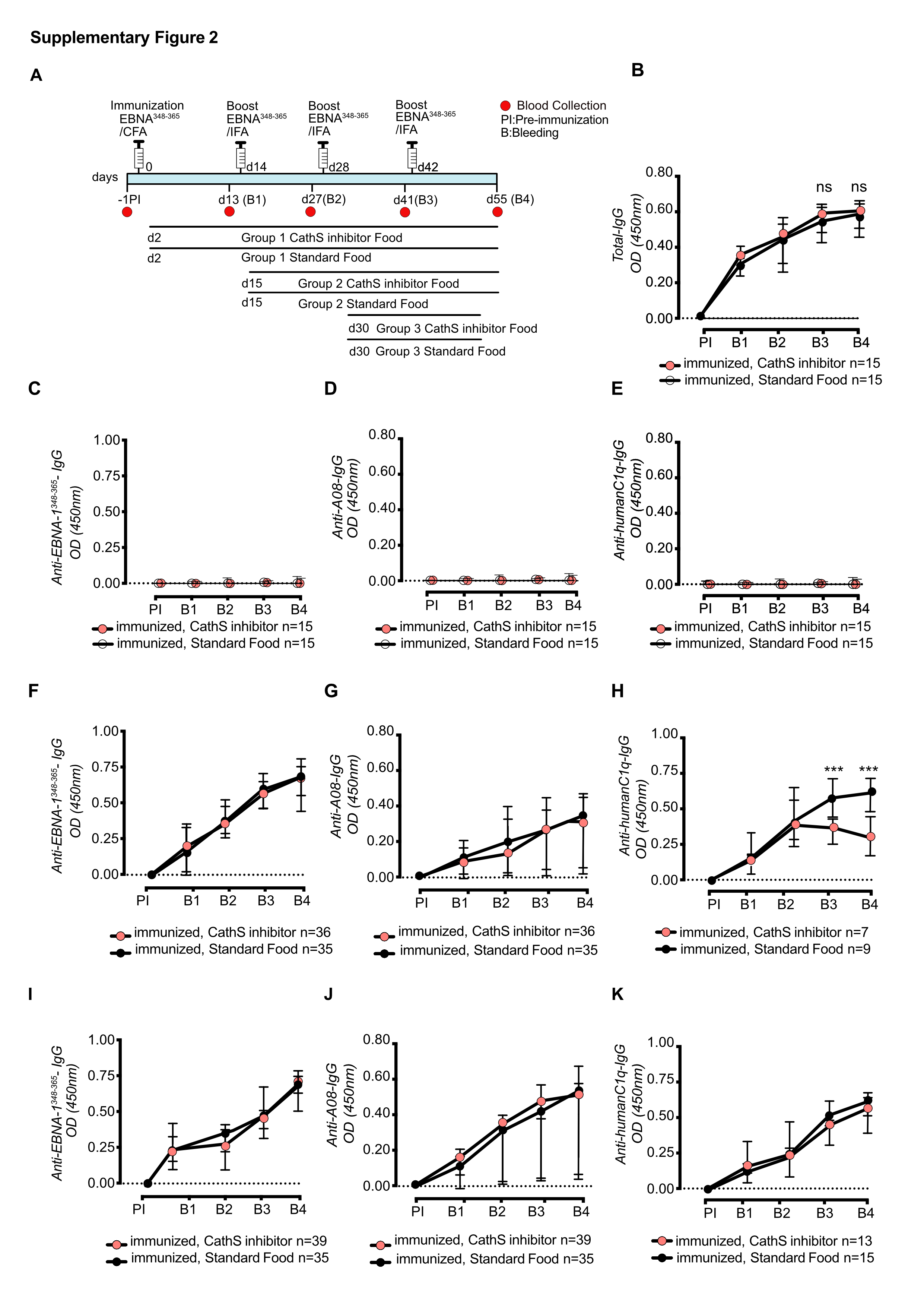

Supplement: Supplementary Figure 2 — CathS inhibitor administration attenuates anti-C1q IgG levels in C1qa-/- mice when administered early, but not at later time points. Immunization schedules and dietary regimen used in three independent cohorts, each randomized to receive standard diet or a CathS inhibitor diet in three different intervention arms (A). Longitudinal analysis of total serum IgG levels in mice immunized with EBNA348–365 peptide and fed either CathS inhibitor diet or standard diet starting at day 15 post-immunization (n = 15/group). (B) Longitudinal analysis of serum, antiEBNA348–365 IgG, anti-A08 IgG, and anti-human C1q IgG in CathS inhibitor diet versus standart diet groups across all cohorts (cohort sizes: n=13–39/group). CathS inhibitor treatment significantly reduced all antibody levels when introduced directly after the start of the immunization schedule (C–E), while there was no effect when introduced late (at day 30) (I–K). In contrast, CathS inhibition significantly reduced anti-C1q levels at B3 and B4 without affecting the primary immunogenicity against EBNA348–365 when started at day 15 of the immunization schedule (F–H) Statistical comparisons were made using repeated-measures ANOVA or two-tailed unpaired t-tests as appropriate; *P < 0.05, **P < 0.01, ***P < 0.001. [file Image2.tiff]

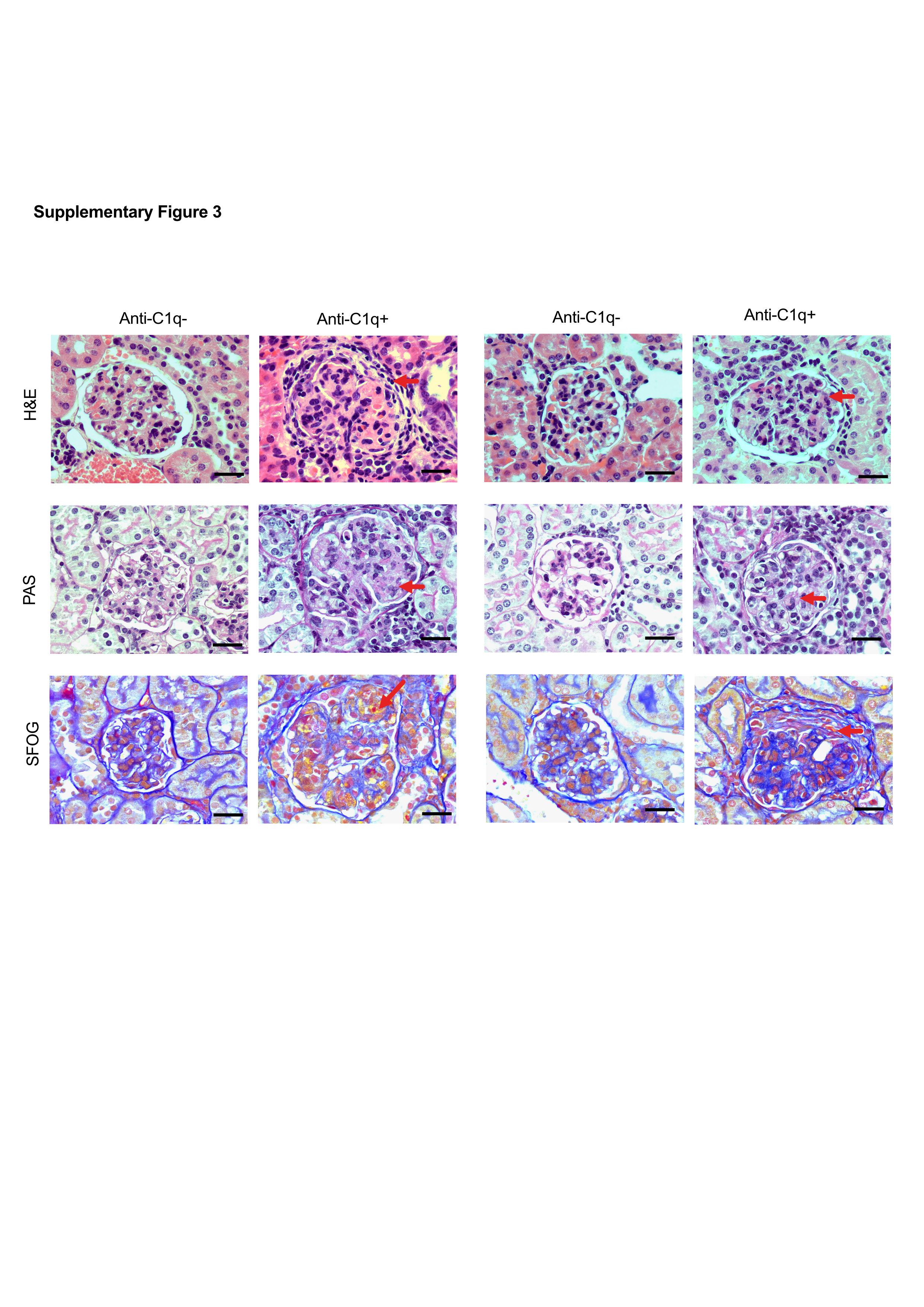

Supplement: Supplementary Figure 3 — Renal histopathology in anti-C1q– and anti-C1q+ mice. Representative glomerular sections stained with H&E, PAS, and Sfog from EBNA348–365 immunized mice grouped according to anti-C1q status. Red arrows indicate glomerular abnormalities including mesangial proliferation (H&E), mesangial matrix expansion and increased cell filtration (PAS), and collagen deposition accumulation. (SFOG)Scale bar = 20 μm. [file Image3.tiff]
